# A Case of Pulmonary Epithelioid Hemangioendothelioma with Literature Review

**DOI:** 10.1155/2020/8048056

**Published:** 2020-10-08

**Authors:** Thu Thu Aung, Andrew Chu, Divya Kondapi, Danny Markabawi, Kanish Mirchia, Pratibha Kaul

**Affiliations:** ^1^Department of Internal Medicine, SUNY Upstate Medical University, Syracuse, New York, USA; ^2^Department of Pulmonary and Critical Care, SUNY Upstate Medical University, Syracuse, New York, USA; ^3^Department of Pathology, SUNY Upstate Medical University, Syracuse, New York, USA

## Abstract

Pulmonary epithelioid hemangioendothelioma is a rare vascular tumor and infrequently described in medical literature as case reports and case series. Diagnosis is often incidental with high index of histopathological suspicion from clinical pathologist. The pathological pattern is quite unique with distinct immunohistochemical stains. Up to this day, there is no established standard treatment owing to the scarcity of this tumor. In this case report, we describe a case of pulmonary epithelioid hemangioendothelioma unexpectedly diagnosed with transthoracic needle biopsy, along with a review of the current literature.

## 1. Introduction

Pulmonary epithelioid hemangioendothelioma (PEH) is a rare vascular tumor of low-grade malignancy potential. It was initially termed intravascular bronchioloalveolar tumor by Dail and Liebow in 1975 [1]. The term “epithelioid hemangioendothelioma” (EHE) was first introduced in 1982 by Weiss and Ezinger to describe a vascular tumor displaying pathological features between hemangioma and angiosacroma [2]. There is no standard of treatment as PEH is extremely rare with only a few case reports and series published in the medical literature. We report a case of a middle age female who had multiple pulmonary nodules on radiographic imaging and subsequently was diagnosed with PEH on transthoracic needle biopsy. A review of literature on PEH with emphasis on histopathologic features will be discussed in this case report.

## 2. Case Report

**Figure 1:**
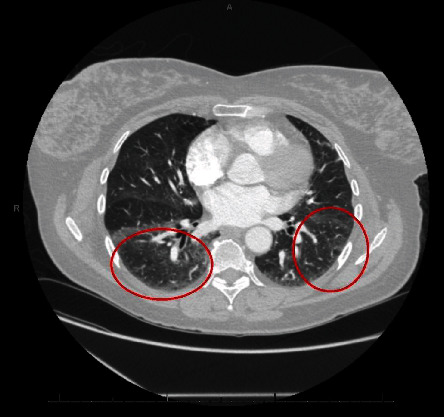
Our patient's initial chest CT scan showing scattered ground glass opacities bilaterally (red circle).

**Figure 2:**
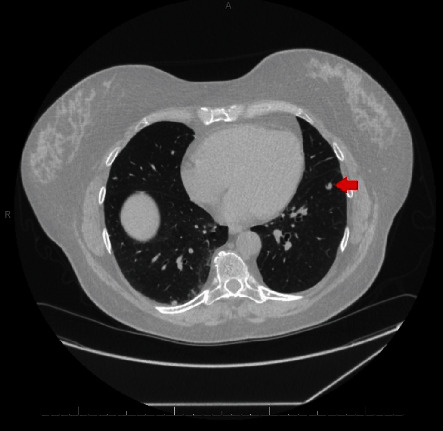
Follow-up chest CT scan showing an 8 mm left lower lobe pulmonary nodule (red arrow) which appeared to be enlarging.

**Figure 3:**
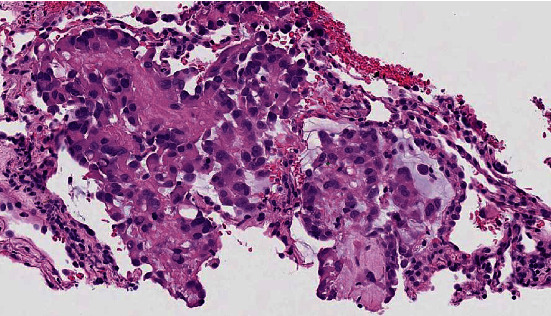
Hematoxylin and eosin stain of transthoracic needle lung biopsy. This slide is at 40x magnification. The sections show multiple cores of lung parenchyma, some of which contain an intra-alveolar infiltrate of relatively monomorphic tumor cells with round nuclei and a moderate eosinophilic cytoplasm. The cells are set in a myxohyaline stroma. Scattered areas show intracytoplasmic lumina, and there are also intranuclear inclusions. Most of the tumor appears to be intra-alveolar and the surrounding lung parenchyma shows mildly prominent type II pneumocytes. There is no evidence of significant nuclear atypia, mitoses, or necrosis.

**Figure 4:**
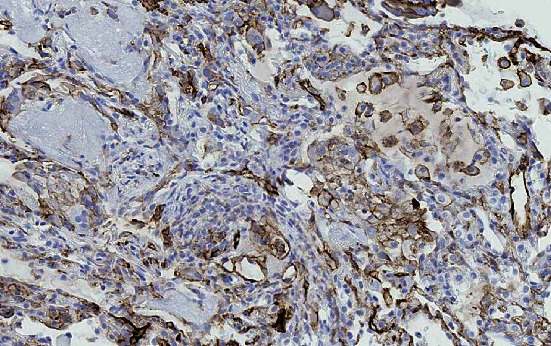
Immunohistochemical stain showing uptake of CD31.

**Figure 5:**
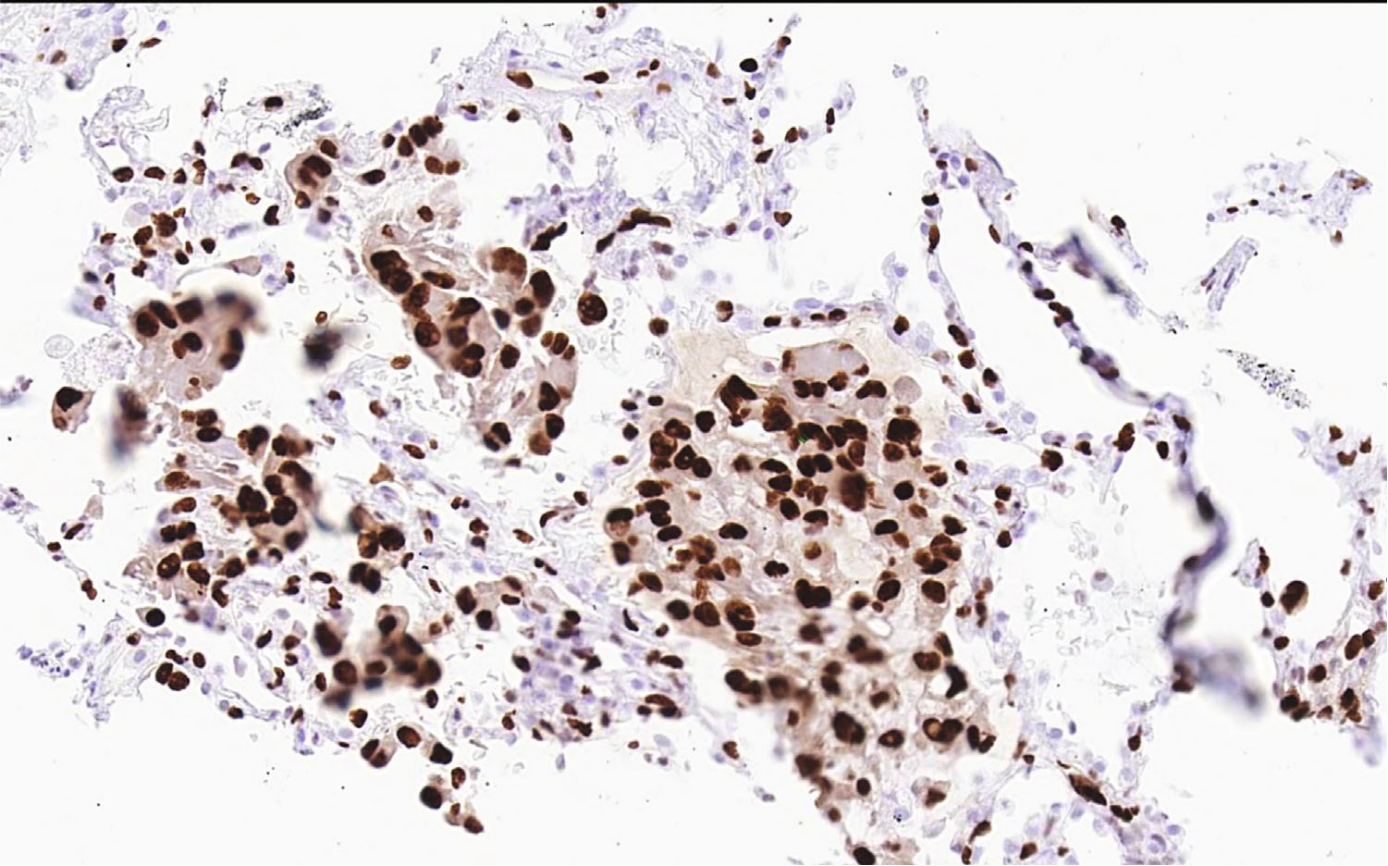
Immunohistochemical stain showing uptake of ERG.

A 58-year-old lady was referred to our clinic for chest tightness and shortness of breath. She had a medical history of subcentimeter pulmonary nodules, peripheral neuropathy, cryoglobulinemia, and polymyalgia rheumatica. She was diagnosed with seronegative Sjögren's syndrome by her rheumatologist about three years ago and was taking hydroxychloroquine, mycophenolate mofetil, and prednisone. She was a nonsmoker. Her vital signs on initial evaluation were stable. She had no wheezing or crackles on physical examination. Pulmonary function tests including spirometry, lung volumes, and diffusion capacity were all within normal range. Computerized tomography (CT) of the chest revealed scattered ground glass opacities bilaterally (Figure 1). She underwent bronchoscopy with transbronchial biopsy which showed mild peribronchiolar fibrosis with negative infectious (bacterial, viral, and fungal) workup. Her respiratory symptoms persisted and she underwent video-assisted thoracotomy and wedge biopsy of right lower lobe. The biopsy showed benign lung tissue with emphysematous changes (airspace enlargement) and focal interstitial and pleural fibrosis. She was managed conservatively. Serial chest CT scans were performed to follow up the pulmonary nodules. On her latest CT scan of the chest, there was an 8 mm left lower lobe pulmonary nodule which appeared to be enlarging (Figure 2). She underwent a positron emission tomography (PET) scan of the whole body which did not reveal any increased standardized uptake value. She subsequently had transthoracic needle biopsy of the enlarging left lower lobe pulmonary nodule. The biopsy revealed an intra-alveolar infiltrate of relatively monomorphic tumor cells with rounded nuclei and moderate eosinophilic cytoplasm. The cells were set in a myxohyaline stroma. Scattered areas showed intracytoplasmic lumina and intranuclear inclusions. Most of the tumor appeared to be intra-alveolar and the surrounding lung parenchyma showed mildly prominent type II pneumocytes. There was no evidence of significant nuclear atypia, mitoses, or necrosis. Immunohistochemical stains showed positive staining of the tumor cells for CD31 and ERG (vascular markers). Pancytokeratin, TTF-1, Pax-8, CAM 5.2, EMA, CDX2, CK20, and CK7 were negative in the tumor cells. The findings fit with a low-grade epithelioid hemangioendothelioma (Figures 3, 4, and 5). She was referred to a large oncology center but no treatment was initiated. The shared decision was made to continue surveillance of the cancer with yearly PET scan. Since then, she has been followed up over a year with no evidence of cancer progression, symptomatically or radiographically.

## 3. Discussion

Since the first pathologic description of EHE by Weiss and Ezinger [2], there have been over 200 cases of PEH published in the literature. The International Hemangioendothelioma, Epithelioid Hemangioendothelioma and Vascular Disorders (HEARD) Registry has the largest collection of data on this rare disease and its natural history [3]. Very few large studies are summarized by Sardaro et al. [4]. The estimated prevalence of PEH is less than one in 1 million cases with female predominance, 4 : 1 ratio. Median age of diagnosis is 36 years old. The liver and lungs are the two most common involved organs. Single organ involvement occurred in more than 60% of the patient. The HEARD Registry observed that the most common EHE involvements are in the liver alone (21%), liver plus lung (18%), lung alone (12%), and bone alone (14%).

Clinical presentation of PEH varies from being asymptomatic to having nonspecific symptoms. Majority of the patients are asymptomatic on initial presentation. A few patients may present with pleuritic chest pain, hemoptysis, and weight loss. Physical examination is often nonspecific, decreased breath sounds due to presence of pleural effusion, or digital clubbing due to persistent hypoxia [5]. The diagnosis of PEH is often made incidentally by radiographic imaging.

Radiograpically, PEH can manifest as three distinct imaging patterns on chest CT: (1) multiple pulmonary nodules with a perivascular distribution; (2) multiple pulmonary reticulonodular opacities; or (3) diffuse infiltrative pleural thickening [6]. The major characteristic radiographic feature of PEH is the presence of perivascular nodules which are usually found near medium-sized vessels and bronchi [7]. The nodules can range up to 3 cm in diameter, but most are often less than 1 cm. Majority of these nodules can be mistaken for metastatic cancer, but PEH shows little or no progression in size on serial CT scans [8]. There is a trend toward better prognosis for multinodular pattern of PEH. In the largest studies reviewing the relation between radiographic patterns and prognosis of PEH, multiple pulmonary nodules on CT scan correspond to have longer survival [5, 9]. However, pleural involvement with malignant pleural effusion is a poor prognostic factor [9, 10].

The diagnosis of PEH is made on the basis of histopathological features and is confirmed using immunohistochemical stain for vascular-endothelial markers. Histology often reveals tumor cells with marked cytologic atypia, centralized stroma with hyalinization, and central necrosis with varying calcification. This central pathology is surrounded by rim of eosinophilic endothelial cells. More severe atypical cytologic such as larger nuclei, prominent eosinophilic nucleoli, and intranuclear cytoplasmic inclusion can also be seen [11, 12]. Histologic finding of spindle tumor cells or fibrinous pleuritic lesion with tumor cell proliferation are linked to worse prognosis [9]. The definitive diagnosis is made with immunohistochemical staining of vascular antigens, such as CD31, CD34, and factor VIII. Other vascular markers can be considered for staining are F8, ERG, EMA, and FKBNP12. A small case series of PEH showed that CD31 and CD34 are the most expressed markers [13].

PEH is an extremely rare entity, and most of the literature is composed of case reports and case series. HEARD registry has been established with the hope that this will lead to more significant study of this rare disease and possibly to evidence-based guidelines for managing it. However, for the time being, we do not have such guidelines on the management of this rare disease. Treatment throughout the literature review has been based on individual basis. Spontaneous partial regression of the cancer has been reported in asymptomatic patients [10]. Cancer surveillance with serial CT scans can be a reasonable option in asymptomatic patients. Surgical resection can be performed in the case of a solitary pulmonary nodule or unilateral multiple pulmonary nodules with no evidence of metastasis [9]. Chemotherapy or immunotherapy can be considered in the cases of disseminated or unresectable disease. There are several cases of cancer regression or remission achieved in patients treated with conventional chemotherapy drugs such as carboplatin, etoposide, and paclitaxel [14]. Since PEH is a tumor of vascular origin, antiangiogenetic drugs such as thalidomide, lenalidomide, and bevacizumab can be considered [15–18]. Several cases have been reported regarding to these antiangiogenetic agents, and some has observed marked response to the treatment [14]. Research and trials are needed to further define the definite role of chemo and immunotherapy.

In our case, the diagnosis of PEH was an unexpected finding on imaging which led to definitive diagnosis with transthoracic needle biopsy. We had high index of suspicion for malignancy despite inconclusive transbronchial and open-lung biopsies because the nodule was enlarging on the chest CT. Since our patient was relatively asymptomatic and no evidence of extrapulmonary manifestation, a shared decision was made between the patient, pulmonologist, oncologist, and radiologist to have yearly follow up of this relatively benign cancer. Should there be a progression of cancer in terms of symptomatology or enlarging size on CT scan, consideration will be given regarding chemotherapy or immunotherapy as discussed above.

## 4. Conclusion

In conclusion, we presented a case of a very rare cancer with no known standardized treatment. Although a registry has been created, we have very little understanding of PEH. This is largely due to the limited number of cases reported and heterogeneity of the disease process. Further studies are needed to provide answers of this unusual neoplasm.
